# Comparison of GeneXpert cycle threshold values with smear microscopy and culture as a measure of mycobacterial burden in five regional referral hospitals of Uganda- A cross-sectional study

**DOI:** 10.1371/journal.pone.0216901

**Published:** 2019-05-15

**Authors:** Irene Najjingo, Winters Muttamba, Bruce J. Kirenga, Joanitah Nalunjogi, Ritah Bakesiima, Francis Olweny, Pastan Lusiba, Achilles Katamba, Moses Joloba, Willy Ssengooba

**Affiliations:** 1 Clinical Epidemiology Unit, School of Medicine, College of Health Sciences, Makerere University, Kampala, Uganda; 2 Lung Institute Makerere University, Kampala, Uganda; 3 Department of Medical Microbiology, College of Health Sciences, Makerere University, Kampala, Uganda; University of Cape Town, SOUTH AFRICA

## Abstract

**Background:**

Determining mycobacterial burden is important in assessing severity of disease, evaluating infectiousness and predicting patient treatment outcomes. Mycobacterial burden assessed by smear microscopy grade and time to culture positivity is clearly interpretable by most physicians. GeneXpert (Xpert) has been recommended by WHO as a first line tuberculosis (TB) diagnostic test as an alternative to smear microscopy. Xpert gives cycle threshold (Ct) values as a potential measure for mycobacterial burden. For physicians to clearly interpret Ct values as measures of mycobacterial burden, this study compared the Xpert quantification capabilities with those of smear microscopy and culture. The study also determined a linear relationship between Xpert Ct values and MGIT culture time to positivity (MGIT-TTP) and associated factors. A cut off Ct value which best predicts smear positivity was also determined using the Receiver Operator Curve analysis method.

**Results:**

Excluding missing results and rifampicin resistant TB cases, a moderately strong correlation of 0.55 between Xpert Ct value and smear grade was obtained. A weak correlation of 0.37 was obtained between Xpert Ct values and MGIT time to positivity while that between Xpert Ct values and LJ culture was 0.34. The Xpert Ct values were found to increase by 2.57 for every unit increase in days to positive and HIV status was significantly associated with this relationship. A cut off Ct value of 23.62 was found to best predict smear positivity regardless of HIV status.

**Conclusion:**

Our study findings show that GeneXpert Ct values are comparable to smear microscopy as a measure of *M*. *tuberculosis* burden and can be used to replace smear microscopy. However, given the low correlation between Xpert Ct value and culture positivity, Xpert Ct values cannot replace culture as a measure of *M*. *tuberculosis* burden among TB patients.

## Introduction

One of the WHO End TB strategies of reducing TB incidence by 80% can be achieved through early case detection[1]. The control measures for TB disease without control of transmission are likely to be less effective because transmission has already occurred to many susceptible contacts by the time a case is properly diagnosed and treated [2].Therefore, contact tracing of possible high transmitters aids early diagnosis of TB even among their susceptible contacts hence stopping continued transmission of TB from infectious cases[3]. Mycobacterial burden, assessed as grades of smear positivity (scanty, 1+, 2+, and 3+) is used to evaluate infectiousness of the patients and assess disease severity. Smear is however less sensitive and requires high bacilli counts which are usually low among HIV-positive TB patients [4]. Time to culture positivity in liquid culture system has for long been used to predict patient treatment outcomes [5]. However, culture is still not readily available due to the long turnaround time that results in patient treatment delays, is prone to contamination and requires specialized infrastructure. Due to these limitations, Xpert has been endorsed by World Health Organization (WHO) as the initial TB diagnostic test among HIV infected individuals[6]. Xpert is a highly sensitive TB diagnostic test with a short turnaround time[7]. It offers quantitative estimation of mycobacterial load in form of cycle threshold values (Ct) which inversely correlates with the concentration of TB bacilli. Low Ct values imply high bacilli load and vice versa. Clinicians are well versed with interpretation of smear grades and time to culture positivity in terms of patient mycobacterial burden[8]. However, it is not clear whether Xpert Ct values could be an effective measure and how the Ct values correlate with smear grades and time to culture positivity.

Patient factors like HIV status and gender have been showed to have an influence on Xpert Ct values [9]. Study site as one of the health system factors has been shown to have an influence on the Xpert Ct values due to the geographical variation in co-infections like HIV. Xpert Ct values have showed a strong correlation with smear grade and time to culture positivity in a study from South Africa, a high TB/HIV-burden setting [10]. However the previous study was done in a research setting, smears were done on frozen sputum samples and only spot samples were used, and this may not be applicable in routine settings. A study concluded that patient delay was not associated with Xpert Ct values [11] yet health system factors like waiting time, study site and distance to health units have been associated with mycobacterial burden [12] [13]. TB has been found to be more prevalent among smokers, people who take alcohol and among people of reproductive age however there has been no study done to evaluate if these could have an influence on Xpert Ct values [14]. We aimed to determine the correlation of Xpert Ct values with smear microscopy grade and culture. Also to assess for a relationship between Xpert Ct values and time to culture positivity for better interpretation of bacterial burden using Xpert Ct value results among patients with TB in Uganda.

## Methods

### Study design and setting

We analyzed data from a cross-sectional study that recruited 1,783 presumptive TB patients from May 2015 to May 2016 of which 495 participants were diagnosed with TB by Xpert. These participants were new and retreatment TB patients above 18 years of age who attended any of the study sites; Mulago, Mbarara, Mbale, Arua and Lacor between May 2015 and May 2016.

### Measurement of mycobacterial burden

Either spot or early morning sputum samples were processed for smear microscopy and Xpert at the field study site as per the standard operating procedures. Smears were graded as scanty, +1, +2 and +3 smear grades. The mean of the five probes (A, B, C, D and E) was used to quantify bacilli by Xpert and the latter reported as mean Ct value. A second sample was shipped to the National TB reference laboratory for culture testing. The sputum was decontaminated using Sodium citrate and Sodium hydroxide (NaOH; 1% final concentration) and neutralized using phosphate buffer saline (PBS; pH 6.8) and concentrated for 15 minutes by centrifugation at 3,00g in a refrigerated centrifuge. The pellet was inoculated in the Mycobacterial growth indicator tube (MGIT) and placed in a MGIT 960 machine for up to six weeks. The pellet was also inoculated on Lowenstein Jensen (LJ) media and incubated with weekly culture reading for up to eight weeks. All positive MGIT cultures were cultured on blood agar to rule-out contamination and confirmed for MTB complex by capilia test. Culture was reported as days to positivity which was the time between sample inoculation into the MGIT tube to the time it flagged positive. We report MGIT machine time to positivity for only cultures with no contamination on blood agar. LJ cultures were graded as no growth, 1–9 colonies reported as actual number, 10–99 colonies graded as +1, 100–200 colonies +2 and >200 colonies as +3.

### Statistical analysis

Descriptive statistics were summarized using proportions for categorical variables, mean or median and interquartile range for continuous variables. Spearman correlation was done to determine the correlation of Xpert Ct values with grades of smear positivity and time to culture positivity as well as LJ culture. The analysis excluded patients who were rifampicin resistant because rifampicin resistance has showed to have delayed PCR amplification and therefore will not have accurate Ct values. In addition a Receiver Operator Curve was used to determine the Ct values that could predict the different levels of smear grade. Linear regression was done to assess the association between MGIT culture time to positivity and Xpert Ct values. First, the assumptions of independence, normal distribution and no collinearity were checked. Variables that had a P-value less than 0.2 were considered for multivariate analysis to assess for interaction and confounding effect on Ct values.

### Ethical consideration

Permission was sought from Makerere University Clinical Epidemiology Unit as well as from the Principal Investigator of the East African Public Health Laboratory Networking Project to use their records. Approval was sought from School of Medicine Research and Ethics Committee (SoMREC).

## Results

Of the 495 individuals who were Xpert positive, 444(89.7%) had LJ culture done and 328 (66.3%) had a MIGIT culture done. Of the 495 Xpert positive patients, 108 were excluded (51 missing Ct values, 51 missing culture, 1 missing smear results and 5 were rifampicin resistant), 387 were analyzed for the correlation of Xpert Ct values with smear microscopy and correlation of Xpert Ct values with LJ culture while 187 were evaluated for the correlation of Xpert Ct values with MGIT culture.

### Socio-demographics and Clinical characteristics of study participants

The distribution of participants by site were; 43(11.1%) from Lacor, 50(12.9%) Mbale, 80(20.7%) Mbarara, 98(25.3%) Arua, and 116 (30.0%) from Mulago. The median age (years; interquartile range) was 32 (27–43) and males were 261 (67.4%). Those who ever smoked in their lifetime were 65 (16.8%) while those who consumed alcohol were 84 (21.1%). Of the 387 participants, 351 (90.7%) were new TB patients and 96 (24.9%) were HIV-positive of whom 76 (79.2%) were on anti-retroviral therapy (ART) as showed in Table 1.

**Table 1:** Socio-demographic characteristics of study participants with pulmonary tuberculosis in Uganda between May 2015 and May 2016.

Variable	Number (n/N) N = 387*	Percentage (%)
**Age** (Categorized at median)		
18–32	198	51.2
33–80	189	48.8
**Study Site**		
Arua	98	25.5
Lacor	43	11.1
Mbale	50	12.9
Mbarara	80	20.7
Mulago	116	30.0
**Sex**		
Males	261	67.4
Females	126	32.6
**Smoke Tobacco**		
Yes	65/383	16.9
No	318/383	82.1
**Alcohol consumption**		
Yes	84/384	21.9
No	300/384	78.1
**History of TB**		
Yes	32/383	8.4
No	351/383	91.6
**HIV status**		
Positive	96/385	24.9
Negative	289/385	75.1
**Whether on ART**		
Yes	76	79.2
No	19	19.8
**Sample type**		
Spot sample	168/382	44.0
Early morning sample	214/382	56.0
**Time to diagnosis (days)** categorized at median		
1–5	197	50.9
6–180	190	49.1

*Missing data

### Distribution of GeneXpert Ct values based history of TB treatment

Those who had no history of TB treatment had a median Ct value; interquartile range (21.78; 15.43–25.13) and those who were retreatment TB cases (relapse, lost to follow up or treatment failure) Ct value; interquartile range (20.22; 16.9–25.24).

### Correlation of Xpert Ct values with smear microscopy grade

**Fig 1:**
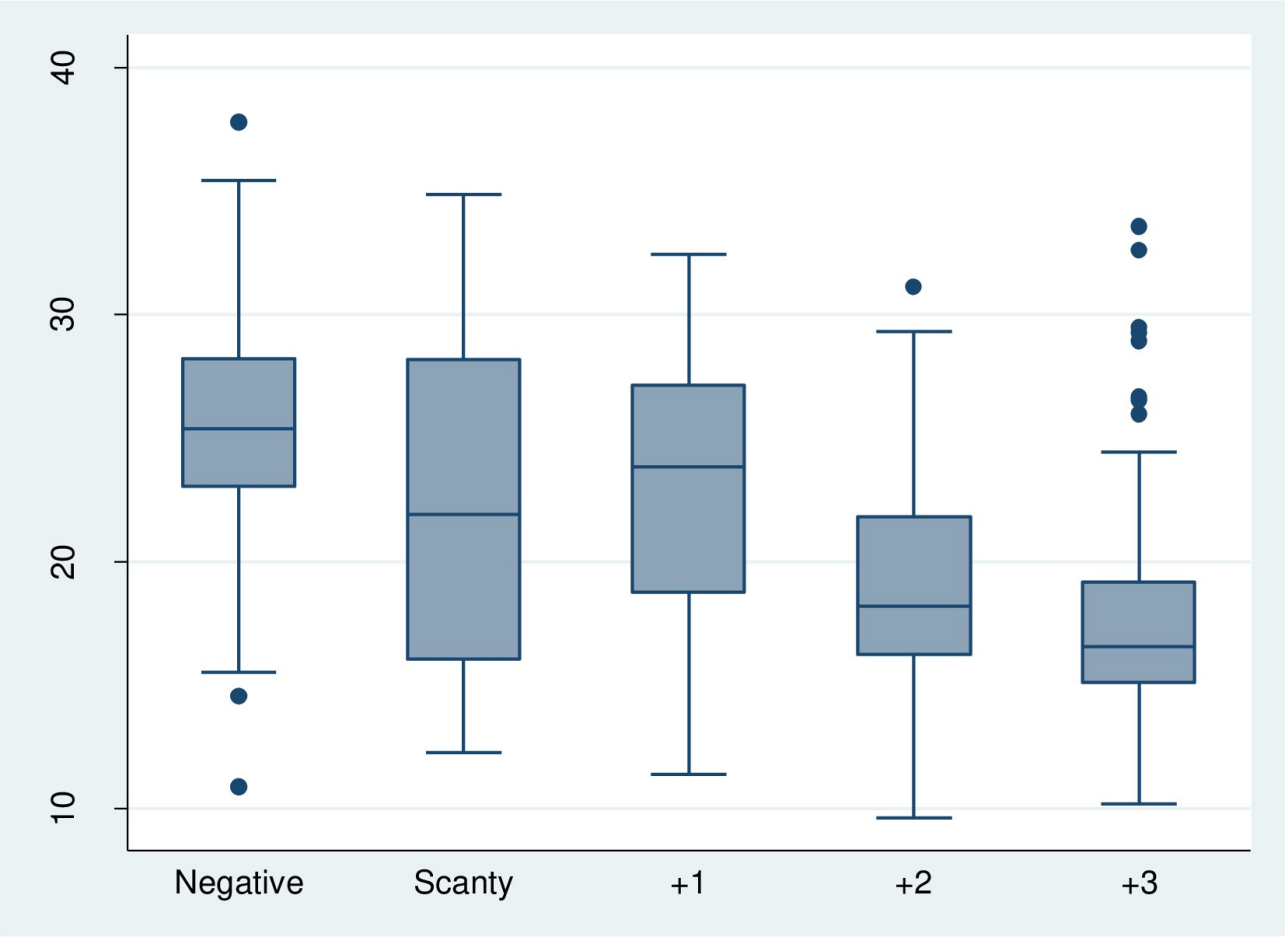
A box plot showing the distribution of the Xpert Ct values with smear grades. A receiver operator curve was used to determine which Ct value could predict smear positivity. A cut off of 23.62 had a sensitivity of 52% and specificity of 72% in predicting +1 smear positivity.

The correlation between the Xpert Ct values and smear microscopy was r = -0.55. There was a decreasing pattern in the Ct values with increasing categories of smear grades. The median Ct values were; 25.4, 23.8, 18.2, 20.1 and 16.6 for negative, scanty, +1, +2 and +3 smear grades respectively as demonstrated with the box plot (Fig 1.).

### Correlation of Xpert Ct values with time to culture positivity

**Fig 2:**
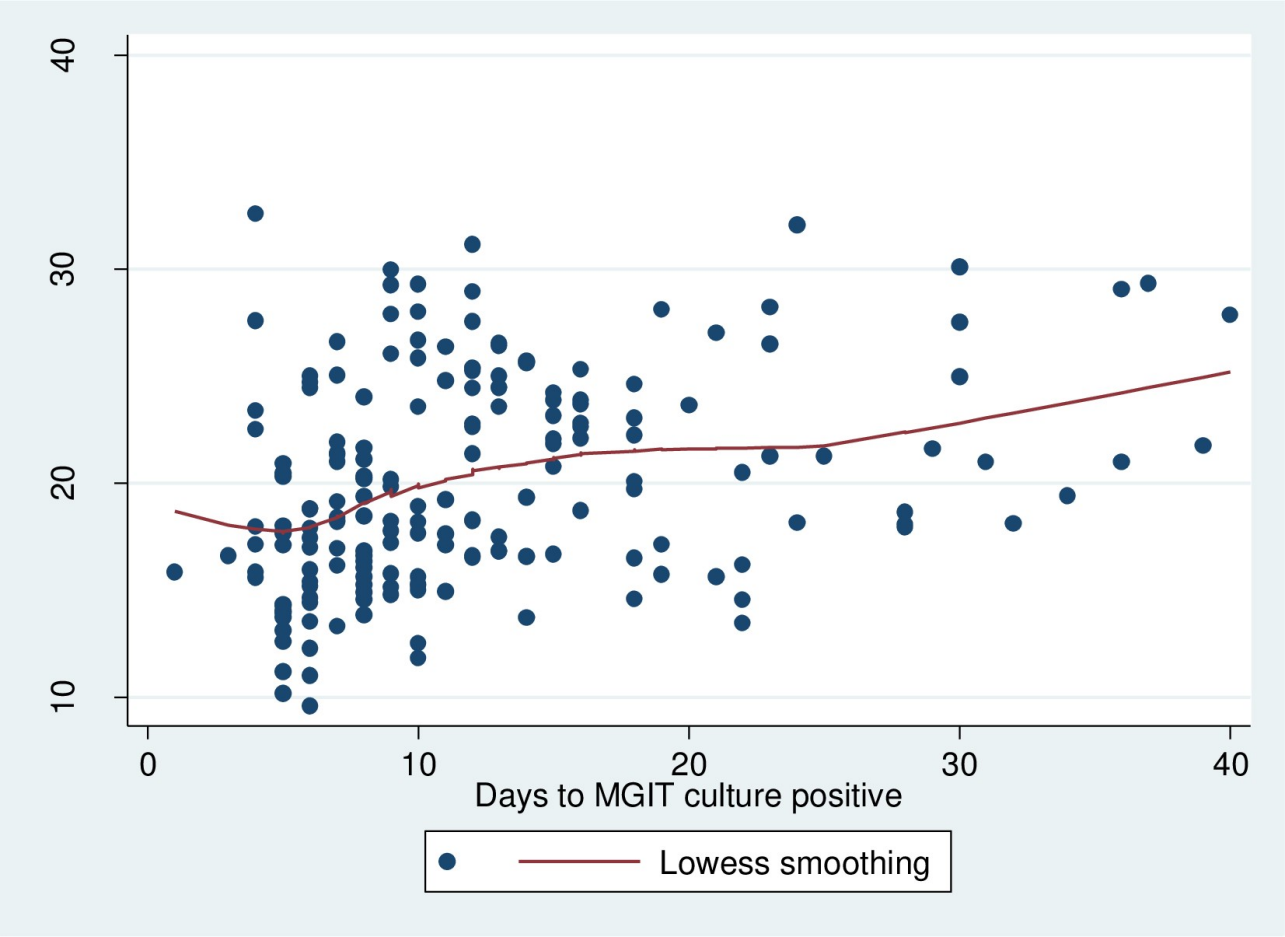
A scatter plot of the Xpert Ct values with time to culture positivity.

There was a correlation of 0.37 between the Xpert Ct values and MGIT time to culture positivity as demonstrated in a scatter plot (Fig 2.). For very low Ct values (>28.01) the median days to positive were 15. Among those who had low Ct values (22.01–28.0) the median days to positive were 13. For individuals with median Ct values (16.01–22.0), the median days to positive were 9. Whereas those with lower Ct values (<16.0), the median days to positive were 7.

### Correlation of Xpert Ct values with LJ culture

There was a negative correlation of r = 0.38 between the Xpert Ct values and LJ culture. There was a decreasing pattern in the median Ct values with increasing categories of LJ culture grades. The median Ct value were; 24.2, 21.4, 19.1, 17.9 and 16.7 for negative, scanty, +1, +2 and +3 LJ culture grades respectively.

### Relationship between Xpert Ct values and time to culture positivity among adults with pulmonary tuberculosis in Uganda

Time to culture positivity was found to have a linear relationship with Xpert Ct values. The Ct values were found to increase by 2.57 for every unit increase in days to MGIT culture positivity.

All variables that had a P-value of less than 0.2 were considered for multivariate analysis as demonstrated in Table 2.

**Table 2:** Bivariate regression of time to culture positivity on Xpert Ct values among adults with pulmonary tuberculosis in Uganda between May 2015 and May 2016.

Variable	Beta coefficient (B)	95% CI	P-Value
**Days to positive**	2.89|	1.76 to 4.02	< 0.001
**Age** (Categorized at median)			
18–32			*Ref*
33–80	-0.17	-1.60 to 1.28	0.819
**Study Site**			
Mbale			*Ref*
Gulu	-0.54	-3.49 to 2.40	0.717
Arua	-0.95	-3.22 to 1.31	0.407
Mbarara	-1.36	-3.70 to 0.97	0.251
Mulago	-2.30	-4.72 to 0.11	0.061
**Sex**			
Females			*Ref*
Males	-1.32	-2.88 to 0.24	0.096
**Smoke Tobacco**			
No			*Ref*
Yes	-1.52	-3.21 to 0.18	0.080
**Alcohol consumption**			
No			*Ref*
Yes	0.04	-0.89 to 0.96	0.08
**History of TB**			
No			*Ref*
Yes	-0.25	-0.99 to 0.49	0.514
**HIV status**			
Negative			*Ref*
Positive	3.06	1.37 to 4.75	< 0.001
**Time to diagnosis (days)**			
1–5			*Ref*
6–180	-1.39	-2.81 to 0.03	0.055

HIV status was the only significant factor associated with Xpert Ct and time to culture positivity values after controlling for distance from health center and site, Table 3. We found the Ct values to increase by 2.34 when individuals with HIV were compared to those without HIV.

**Table 3:** Multivariate analysis of the time to culture positivity on Xpert Ct values among adults with pulmonary tuberculosis in Uganda between May 2015 and May 2016.

Variable	Beta coefficient (β)	95% CI	P-Value
**HIV status**			
Negative			*Ref*
Positive	2.34	0.70 to 3.99	0.005
**Days to positive**	2.57	1.44 to 3.71	< 0.001

## Discussion

Among the TB patients from 5 regional referral hospitals in Uganda, we found Xpert Ct values comparable with smear microscopy to a minimal extent and with culture to a smaller extent in assessing mycobacterial burden. A cut off of 23.62 had the highest sensitivity and specificity in predicting +1 smear grades respectively. We also found a linear relationship between Xpert Ct values and time to culture positivity, HIV status was associated with this relationship. Xpert Ct values were found to increase by 2.23 when HIV positive individuals were compared with HIV negative individuals.

The negative correlation of 0.55 between the Xpert Ct values and smear grades was in the range of the different correlations observed in South Africa [9]. In South Africa, Hanrahan observed correlations between 0.54 to 0.74, however these differed depending on immunosuppression of the patients. A study by Blakemore observed a correlation of 0.77 which was higher than that observed in our study. This could be because in the previous study, three samples were collected and two samples were processed which could have increased the concentration of the bacilli load in the sample. In the current study, we collected one sample, processed and used for the testing [10].

In addition our study findings are similar with a study by Theron that compared Xpert Ct values with smear and culture and found low correaltion dispalyed in reduced sensitivity between these diagnostic tests. Theron found that Xpert outperformed both smear and culture as many patient were positive for Xpert yet negative on culture and smear[15].

We found stronger correlations among those with high Ct values and high smear grades compared to those with low Ct values and low smear grades. This is possibly because the log transformation of colony forming units in a 2+ smear grade is not different to that of a 3+ smear grade. This finding is similar to that from a multisite study which showed overlap in the Ct values in the different smear grades [10]. A Ct value of 23.62 was found to have a sensitivity and specificity of 52% and 72% respectively in predicating +1 smear grade. This is similar to findings that showed that a Ct value of 25.0 had the highest sensitivity and specificity of 95% and 65% respectively in predicting smear positivity [10].

There was a weak correlation of 0.37 between Xpert Ct values and MGIT time to positivity. This correlation was not much different from that observed in South Africa. Hanrahan observed correlations that varied from 0.41 to 0.64 which differed by level of immunosuppression [9]. This correlation of 0.37 observed in our study was however weaker than that of 0.68 which was observed by Balkamore [10]. In the previous study, two samples were collected, processed and inoculated in culture media and in the Xpert cartridge. In our study, two samples were collected, one directly used for smear microscopy and Xpert and the second sample processed for culture. Performing culture and Xpert on different samples may have influenced this difference, however, since the type of the sample, spot or early morning was rondom, this may have just minimal influence. In addition a study by Prakash that was acessing clinical utility of Xpert Ct values in diagnosing TB showed that the correlation between Xpert and culture would aid predict bacilli load however pointed out the reduced sensitivity especially for samples with very low bacilli load. The sensitivity between the Xpert Ct values and culture was stronger among those with high bacilli (low Ct values) load compared to those with low bacilli load (high Ct values) [16].

The median days to positive were 16 among those who had very low Ct values, 13 days among those who had low Ct values, 9 days for individuals with median Ct values and 7 days for those with high Ct values.

HIV positivity was found to be associated with high Xpert Ct values and time to culture positivity. There was a 2.34 unit increase in Ct values among the HIV positive individuals compared to the HIV negative individuals. This is consistent with studies that have found high Ct values among HIV positive individuals and low Ct values among HIV negative individuals. Studies have reported poor cavitation among the HIV positive individuals hence having low burden of TB [10].

Patient delay was not associated with reduced or improved performance of Xpert Ct values. These findings are similar to findings from a cross-sectional study by Ssengooba which found that patient delay was not associated with performance of Xpert Ct values. The former study had a smaller sample size but even with adequate sample size, patient delay which was assessed with time from symptom to diagnosis was found not to be associated with this correlation thus patient delays may have no effect on the correlation of Xpert Ct values with smear or culture [11,17]. Sex was not associated with performance of Xpert Ct values and these findings are similar to what Beynon got and found sex not associated with performance of Ct values [18]. Our findings are further supported by a study by Hanrahan who found sex not to be significantly associated with performance of Ct values even when there was a difference in the mean Xpert Ct values among men and women hence sex has no effect on Xpert Ct values and correlation. Site was not associated with Xpert Ct values yet a study by Balkamore found an association by site. In the present study, samples were collected from the different sites and cultured at the National TB reference laboratory hence no difference in culture results. For smear grading, the sites were regional referral hospitals and used similar grading system which could have increased uniformity in results reporting.

### Strength of the study

The study was done in five main regional referral hospitals well spread in Uganda which makes the study findings generalizable. The study had adequate sample size to determine the correlation of the Xpert Ct values and smear microscopy which makes the findings authentic. This is one of the first studies for correlation in this setting which makes it unique in terms of knowledge. Standard operating procedures and WHO guidelines followed when collecting and processing samples which improves on the credibility of the results. The study was done in a clinical setting which represents the true outcomes of the testing.

### Limitations

There was missing data on MGIT culture which could have affected correlation of xpert Ct values with time to culture positivity. There were few numbers in some subgroups due to missing culture results, making it difficult to detect a meaningful difference in association with these variables. One sample was used for susbequent analysis yet other studies have used more than one sample hence reduced concentration of viable TB bacilli.

## Conclusion

GeneXpert cycle threshold values provide a moderate measure of bacilli load in comparison with smear microscopy. Majority of low and very low Ct values quantified by Xpert were negative by smear microscopy which poses to the community a risk of getting TB. This is because of the ability of the smear negative but Xpert positive individuals to transmit TB has been found to be significant. In line with the WHO policy that recommends use of Xpert as the initial TB diagnosis test among HIV positive individuals, Xpert Ct values can be used as a measure of bacilli load among HIV-positive individuals.

Due to the weak relationship between Xpert Ct values with time to culture positivity, these tests cannot be used in relation to another as independent predictors of bacilli burden. Therefore studies with sufficient sample size should be carried out to assess this relationship.

## Supporting information

S1 Dataset Data set attached in excel format. (XLS)
